# Detecting implicit and explicit facial emotions at different ages

**DOI:** 10.1007/s10433-024-00805-1

**Published:** 2024-03-19

**Authors:** Giulia Prete, Irene Ceccato, Emanuela Bartolini, Adolfo Di Crosta, Pasquale La Malva, Rocco Palumbo, Bruno Laeng, Luca Tommasi, Nicola Mammarella, Alberto Di Domenico

**Affiliations:** 1 Department of Psychological, Health and Territorial Sciences, “G. d’Annunzio” University of Chieti-Pescara, 31, Via Dei Vestini, 66013 Chieti, Italy; 2 Department of Neuroscience, Imaging and Clinical Sciences, “G. d’Annunzio” University of Chieti-Pescara, Chieti, Italy; 3 Department of Medicine and Aging Sciences, “G. d’Annunzio” University of Chieti-Pescara, Chieti, Italy; 4 Department of Psychology, University of Oslo, Oslo, Norway

**Keywords:** Go/no-go task, Emotion processing, Spatial frequencies, Hybrid faces, Age-related differences

## Abstract

Emotions are processed in the brain through a cortical route, responsible for detailed-conscious recognition and mainly based on image High Spatial Frequencies (HSF), and a subcortical route, responsible for coarse-unconscious processing and based on Low SF (LSF). However, little is known about possible changes in the functioning of the two routes in ageing. In the present go/no-go online task, 112 younger adults and 111 older adults were asked to press a button when a happy or angry face appeared (go) and to inhibit responses for neutral faces (no-go). Facial stimuli were presented unfiltered (broadband image), filtered at HSF and LSF, and hybrids (LSF of an emotional expression superimposed to the HSF of the same face with a neutral expression). All stimuli were also presented rotated on the vertical axis (upside-down) to investigate the global analysis of faces in ageing. Results showed an overall better performance of younger compared to older participants for all conditions except for hybrid stimuli. The expected face-inversion effect was confirmed in both age groups. We conclude that, besides an overall worsening of the perceptual skill with ageing, no specific impairment in the functioning of both the cortical and the subcortical route emerged.

## Introduction

Facial emotions constitute the most critical cue in non-verbal communication, so two different cerebral routes have evolved to specifically cope with their analysis (LeDoux 1996): the cortical route includes the Fusiform Face Area and other temporal and frontal regions, and it is responsible for detailed, conscious, but relatively slow processing of facial stimuli; the subcortical route includes the amygdala, the pulvinar and the superior colliculus (Morris et al. 1999) and it is responsible for a fast but unconscious analysis (Johnson 2005). Different studies revealed that each route is specialised in encoding specific features of the stimuli, with facial sex and identity preferentially processed by the cortical route, primarily based on the analysis of the High Spatial Frequencies (HSF) of the stimulus, and with emotional expressions preferentially processed through the subcortical route, which is primarily based on the analysis of the Low Spatial Frequencies (LSF) of the image (Lacroix et al. 2021; Vuilleumier et al. 2003; Williams et al. 2004). Interestingly, some evidence also suggests an association between different ranges of spatial frequencies and specific emotional expressions (Cassidy et al. 2021; Kumar and Srinivasan 2011), making the field of the cortical/subcortical correlates of facial expressions even more complex.

A crucial question often overlooked in this domain is the role of ageing and the possible changes in the lifespan concerning the role of the two cerebral routes during the processing of emotional stimuli. In the visual domain, the importance of the LSF (vs. HSF) has already been demonstrated in the foetus (Reid et al. 2017) and in newborns (Johnson 2005). Still, it has also been found that in infants, emotional processing seems to be mainly based on the HSF (van den Boomen et al. 2019). However, contrasting evidence has been found (Pellicano and Rhodes 2003), showing a non-linear relationship between age and the role of different SF/global vs local analysis in emotion detection. In this context, little is known about the possible effects of ageing: for instance, it has been found that older participants show a decrease in performance, compared to younger participants, specifically for HSF scenes (no difference emerged for LSF images), in an indoor vs outdoor categorisation task (Ramanoël et al. 2015), but also that the global analysis of faces does not change across ages (Boutet and Faubert 2006). Importantly, regarding emotions, a “positivity bias” has been widely documented in older adults (Mammarella et al.2016a, 2017; b; Reed et al. 2014). This bias consists of a lower impact of negative information on attention and memory processes in older adults than in younger adults (Mammarella et al. 2016b; Mather 2016; Mather and Knight 2005), with less accurate performance in recalling negative events (Charles 2010; Ceccato et al. 2022). This bias has been explained as due to the fact that when the temporal horizon of persons is limited, they would try to avoid negative emotions and would be strongly oriented toward positive experiences (Carstensen et al. 2006; Fusi et al. 2022; Cannito et al. 2021): this would happen in ageing when residual life duration is usually perceived as progressively limited (Ceccato et al. 2021, 2023). This positivity bias has been recently confirmed with surprised faces, considered ambiguous in valence (Barber et al. 2022). The authors found that older adults judged LSF and HSF surprised faces less negatively than younger adults. They proposed that, with ageing, a weaker activity of the subcortical route makes the cortical activity more influential in driving the response, which shows the expected positivity bias due to top-down cognitive control. This idea has been recently confirmed in a neuroimaging study showing that the positivity bias in older adults might be related to stronger activity in prefrontal areas (cortical route) and in reduced spontaneous activity of the amygdala (subcortical route), which is deactivated through a top-down cortical-subcortical neural pathway (Petro et al. 2021).

In this domain, “hybrid faces” constitute an interesting tool to investigate the relationship between SF and emotion processing through the two cerebral routes: they are created by superimposing the image of an emotional face filtered at LSF to the image of the same face with a neutral pose filtered at HSF. In this way, it is possible to investigate how the subcortical route (emotions shown in LSF) and the cortical route (neutral expression shown in HSF) work and how the brain processes the contrasting information in the same image. Hybrid stimuli were initially proposed by Schyns and Oliva (1994) and then they were modified and differently proposed by Laeng and colleagues (Laeng et al. 2010): in their version, a hybrid stimulus contains an emotional image filtered at 1-to-6 cycles per image (cpi), which is superimposed to the image of the same actor expressing a neutral pose and filtered at middle and high spatial frequencies (7–178 cpi). By using this set of stimuli, Laeng and colleagues (2010) showed that even if participants were not able to explicitly label the emotions hidden in the LSF of hybrid stimuli (i.e., all stimuli were labelled as “neutral”), hybrid faces containing happy expressions were systematically judged as more friendly than those containing angry expressions. The authors concluded that the activity of the subcortical route is effectively involved in the unconscious processing of emotions, leading to an implicit modulation of emotional judgments. This speculation was also supported by the evidence that, differently from healthy controls, a patient with a lesion in the subcortical route (including the amygdala) did not show the expected friendliness judgment modulation according to the LSF of hybrid stimuli (Laeng et al. 2010). Starting from this last evidence, several further studies exploited the same hybrid stimuli as those used by Laeng et al. (2010) to investigate subliminal emotion processing, both at a behavioural (Prete et al. 2014, 2015b, 2018; c) and electrophysiological level (Laeng et al. 2013; Prete et al. 2015a, 2019), confirming the implicit modulation of emotional processing through the LSF of the images (Neta et al. 2021). The advantage of such a category of stimuli in the study of subliminal emotions is that they can be presented for a long time, thus avoiding the need for a very brief presentation (i.e., tachistoscopic paradigm; e.g., Wang et al. 2021). They ensure that the emotional content remains under the level of awareness, given the possibility to indirectly measure the activation of the subcortical route.

Furthermore, besides being associated with emotional processing, the LSF of the image are also related to the global analysis of the stimuli (e.g., spatial orientation), as opposed to the HSF, mainly involved in the processing of local elements (e.g., details of a specific face). The well-known “face-inversion effect”, namely the systematic decrement in the ability to process facial details (local elements) when faces are rotated 180° about their vertical axis (Diamond and Carey 1986), confirms that local (e.g., mouth and eyes) and global (orientation) analysis of faces are distinctive processes (Psalta et al. 2014). It has been shown that emotional expressions are related to the global processing of stimuli (Psalta and Andrews 2014). Thus, we can conclude that facial emotions are rapidly processed by the subcortical route, which is responsible for an implicit, fast, and unconscious analysis, based on the global processing of the percept, mainly based on the LSF.

Starting from this complex frame, the present study aims to shed light on the functioning of the subcortical route in ageing, investigated by exploiting hybrid faces as in previous studies (Laeng et al. 2010; Prete et al. 2014, 2015b, c, 2018; Laeng et al. 2013; Prete et al. 2015a, 2019). Differently from previous evidence collected with hybrid stimuli, in which participants were asked to express a friendliness judgment, here a go/no-go task is used to quantify the explicit categorisation of emotional versus neutral faces presented as unfiltered (broadband), filtered at HSF, filtered at LSF and hybrid. Two age groups (younger and older adults) were tested in an emotional go/no-go task to directly compare the performance at different ages. Moreover, to further investigate the relationship between global/local analysis and the functioning of the subcortical/cortical route, all stimuli were presented in canonical (upright) and inverted orientation (upside-down). We asked participants to detect the emotional stimuli (go) and to avoid a response when neutral faces were presented (no-go). We expected a better performance in younger adults than older adults for faces filtered at LSF and hybrid (subcortical route) due to a weakening activity of the subcortical route in ageing (Barber et al. 2022; Petro et al. 2021). For the same reason, a weaker performance was hypothesised in older compared to younger participants for stimuli presented upside-down (global analysis based on the LSF). Instead, an absence of age difference was expected for stimuli presented both unfiltered and filtered at HSF since these stimuli would activate the cortical route, which is supposed to be strongly active also in ageing (Petro et al. 2021). Finally, in accordance with the positivity bias, better performance in ageing was expected for positive (i.e., happy) than for negative (i.e., angry) facial expressions (Barber et al. 2022; Di Domenico et al. 2015; Mather 2016). Data collected through a go/no-go emotional task were analysed to show how each of the factors manipulated in the study interacted with each other, and we expected younger participants to outperform older ones for LSF and hybrid stimuli, mainly when presented in inverted orientation, particularly with an angry expression.

## Materials and methods

### Participants

Participants were contacted via social media messages and emails sent to university students, who were asked to also involve relatives and friends. Students were informed that, for whom would be interested in, the results collected for this study would be discussed together in classroom (they did not receive payment for participation). Exclusion criteria were explicitly stated in the initial messages, consisting in neurological and/or psychiatric conditions, as well as visual impairments. Considering possible dropout, which is high in online studies, we decided to stop the administration when at least 130 younger and 130 older participants completed the paradigm, with the aim to obtain a final sample of at least 100 participants in each age group. From the initial sample of 277 participants, 41 responders started the task online, but they did not complete the paradigm, 6 younger and 7 older participants were excluded from the analyses because their accuracy means were two standard deviations out than the total mean of their age group. A final sample of 223 participants was included in the analyses, divided into two groups: 112 younger adults (YA), including 79 females and 33 males, with an age between 18 and 35 years (mean age ± standard error: 24.27 ± 0.37 years old), including 10 left-handers, as self-reported; 111 older adults (OA), including 64 females and 47 males, with an age between 65 and 94 years (72.84 ± 0.71 years-old), including 2 self-reported left-handers. Prior the beginning of the task, all participants declared their handedness, to have normal or corrected-to-normal vision, as well the absence of neurological and/or psychiatric conditions. All were unaware of the purpose of the study.

### Stimuli

**Fig. 1:**
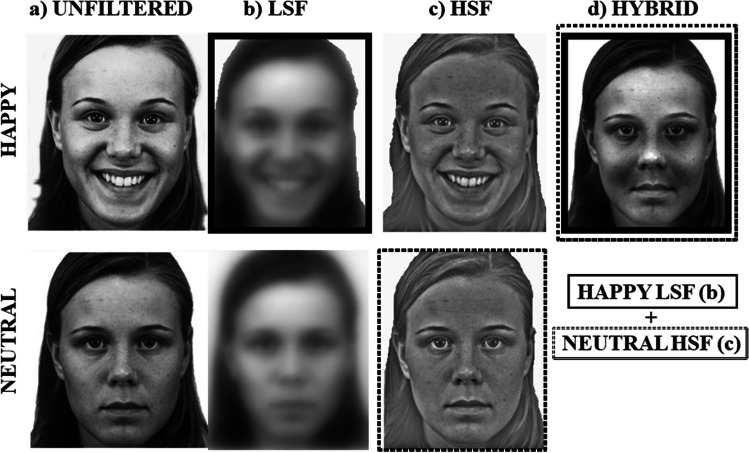
Examples of happy (upper row) and neutral (lower row) faces presented as: **a** unfiltered (e.g., original stimuli), **b** filtered at Low Spatial Frequencies (LSF: 1–6 cpi), **c** filtered at High Spatial Frequencies (HSF: 7–128 cpi), and **d** hybrid faces, created by superimposing the LSF of an emotional face (in this case, happy LSF stimulus, black frame) to the HSF of the neutral expression of the same face (in this case, neutral HSF stimulus, dotted frame). Neutral hybrid faces were the same as neutral unfiltered stimuli (neutral LSF superimposed to neutral HSF)

Stimuli were created from photographs in the Karolinska Directed Emotional Faces (Lundqvist et al. 1998), a database of faces in neutral and emotional poses. Photographs in frontal view of 15 female and 15 male faces in happy, angry, and neutral expressions were selected, converted into grey-scale images, measuring 5.2° × 5.3° of visual angle (260 × 270 pixels) seen at a distance of 72 cm (see Fig. 1a). Then, all stimuli were filtered through MatLab software (Mathworks Inc., Natick, MA), obtaining one image filtered at low spatial frequency (LSF; 1–6 cycle per image: cpi; see Fig. 1b) and another image filtered at high spatial frequency (HSF; 7–128 cpi; see Fig. 1c). Experimental stimuli were constituted of unfiltered faces, faces presented at LSF, at HSF, and hybrid faces. Emotional hybrid faces were created by superimposing the LSF of an emotional face (either angry or happy) to the photograph in HSF of the same face with a neutral expression (see Fig. 1d). Hybrid neutral faces were presented unfiltered (i.e., neutral LSF was superimposed to neutral HSF, resulting in a neutral broadband image; see (Prete et al. 2018; Tommasi et al. 2021).

### Procedure

Once recruited, participants were invited to read and subscribe an informed content to take part in the study, and then to download on their PC an E-Prime Go auto-run script. At the beginning, they were instructed to carry out the task in isolation and in a dark and silent room, and to position the screen monitor at 72 cm from their face (auto-run did not work on tablets and smartphones, indeed the task can be run only on PC). Stimuli were presented on a white background in the centre of the screen (1024 × 768 pixels). Participants were instructed to avoid any movement and to maintain their position, and they performed two sessions of 480 trials, one “happy” and one “angry” session, which order was balanced among participants. In a session, each of the 30 identities was presented in a neutral and emotional pose (either angry or happy in the two sessions), and each of these 60 stimuli was presented unfiltered, filtered at LSF, filtered at HSF, and hybrid. The 240 stimuli were presented upright (canonical orientation) and upside-down (rotated 180° on the vertical axis) for a final set of 480 different trials in the Angry session and 480 different trials in the Happy session (no repetition of the same stimulus in the same orientation was included). Happy and angry expressions were presented in two different sessions to make the task easier especially for older participants, and for the same reason a Go/No-Go task was created, in order to have just one response key and a easy-to-be-remembered task (i.e., detect a target expression).

**Fig. 2:**
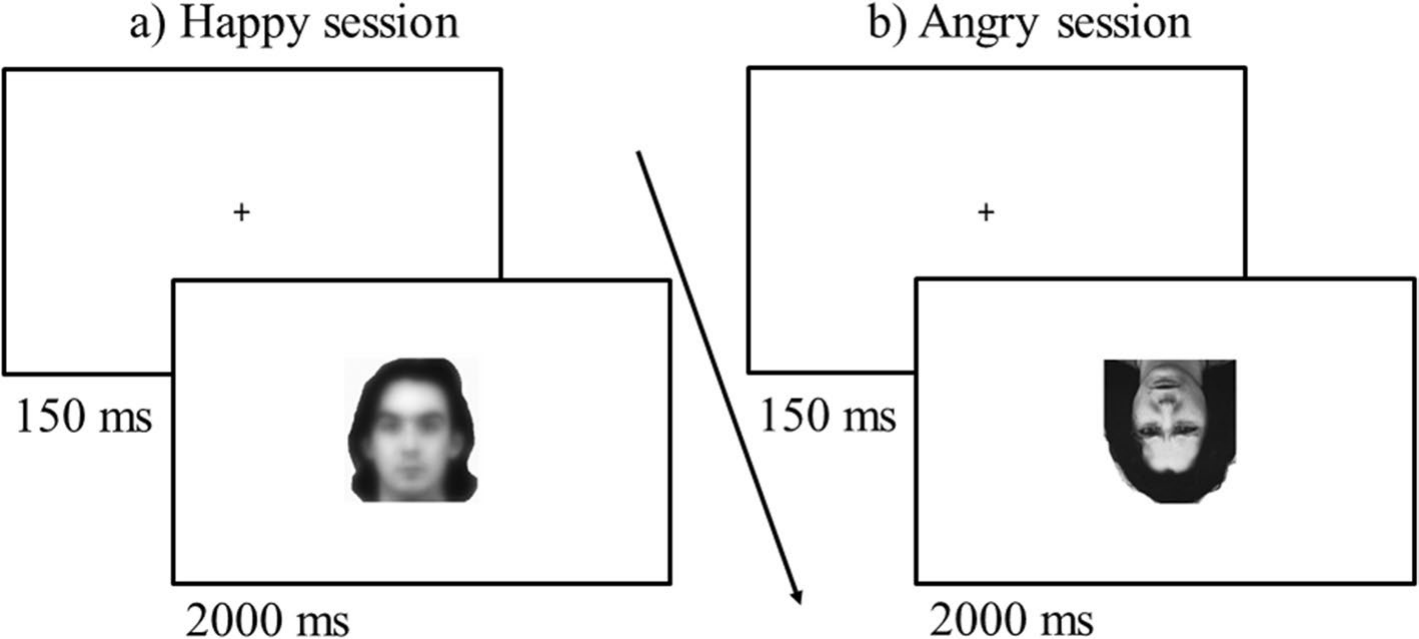
Schematic representation of the two sessions of the paradigm: **a** neutral face presented upright at LSF (example of a no-go trial); **b** angry face presented upside-down unfiltered (example of a go trial)

In each trial, after a fixation cross presented in the centre of the screen for 150 ms, a stimulus was presented for 2 s, during which the participant was required to give their response, otherwise the next trial started (after 2 s, which was a fixed presentation time). Participants were instructed to detect the target emotional expression, disregarding both filtering and spatial orientation, by pressing the key “m” on the keyboard as soon as an emotional face appeared (either angry or happy in the two separate sessions), otherwise they were asked to give no response when a neutral face was presented (with neutral stimuli constituting 50% of the trials; see Fig. 2). The presentation order of the trials was randomised within and across participants in each session. A set of 8 trials was presented before the beginning of the task to allow participants to familiarise themselves with the procedure.

All participants had given informed consent to participate in the research. The procedure was carried out following the principles of the Declaration of Helsinki and was approved by the Institutional Review Board of Psychology of the [*anonymized for review*] (protocol number: [*anonymized for review*]). The paradigm lasted about 30 min and was shared and controlled by E-Prime-go software (Psychology Software Tools, Inc., Pittsburgh, PA).

### Statistical analysis

Data were automatically recorded on the E-Prime Go server and were then downloaded and aggregated. For each participant, the proportion of false alarms (wrong detection of an emotional expression when a neutral stimulus was presented) was subtracted from the proportion of hits (correct detection of the emotional trials) to obtain the overall index of the performance Pr (i.e., Pr = Hits—False alarms; Snodgrass and Corwin 1988). Data were analysed by using Statistica software (StatSoft Inc., Version 7), employing a 2 × 2 × 2 × 4 analysis of variance (ANOVA), using Pr as the dependent variable, Group (YA, OA) as a between-subject factor, and Emotion (Angry, Happy), Orientation (Upright, Upside-down) and Filtering (Unfiltered, LSF, HSF, Hybrid) as within-subject factors. When needed, post-hoc comparisons were computed by using the Duncan test, and the significance threshold was set at *p* = 0.05.

## Results

All results are reported in Table 1. Main effects were all significant: Group (*F*_(1, 221)_ = 40.021, *p* < 0.001, *η*^*2*^_*p*_ = 0.153) showed a better performance of YA (0.627 ± 0.013) compared to OA (0.521 ± 0.02); Emotion (*F*_(1, 221)_ = 191.01, *p* < 0.001, *η*^*2*^_*p*_ = 0.464) showed higher Pr scores for Happy (0.635 ± 0.013) than for Angry faces (0.514 ± 0.013); Orientation (*F*_(1, 221)_ = 116.88, *p* < 0.001, *η*^*2*^_*p*_ = 0.346) confirmed the expected better performance for Upright (0.604 ± 0.012) than for Upside-down presentation (0.544 ± 0.014). Also, Filtering was significant (*F*_(3, 663)_ = 2578.02, *p* < 0.001, *η*^*2*^_*p*_ = 0.921). Post-hoc comparisons revealed that Pr was lower for Hybrid (0.046 ± 0.007) compared to all of the other filtering conditions and for LSF (0.677 ± 0.017) compared to both HSF (0.782 ± 0.014) and Unfiltered (0.793 ± 0.014) stimuli (*p* < 0.001 for all comparisons).

**Table 1:** Results of the 2 × 2 × 2 × 4 ANOVA (Group, Emotion, Orientation, Filtering) on the Pr score

	*MS*	*F*	*p*	*η*^*2*^_*p*_
Group	9.963	40.021	< 0.001	0.153
Emotion	13.097	191.010	< 0.001	0.463
Emotion × group	0.020	0.291	0.590	0.001
Orientation	3.229	116.878	< 0.001	0.346
Orientation × group	1.009	36.539	< 0.001	0.142
Filtering	112.988	2578.019	< 0.001	0.921
Filtering × group	1.604	36.595	< 0.001	0.142
Emotion × Orientation	0.599	58.570	< 0.001	0.210
Emotion × orientation × group	0.133	13.032	0.0003	0.056
Emotion × filtering	0.918	60.427	< 0.001	0.215
Emotion × filtering × group	0.047	3.101	0.026	0.014
Orientation × filtering	0.632	80.214	< 0.001	0.266
Orientation × filtering × group	0.106	13.494	< 0.001	0.058
Emotion × orientation × filtering	0.219	32.844	< 0.001	0.129
Emotion × orientation × filtering × group	0.087	12.973	< 0.001	0.055

The interaction between Group and Filtering (*F*_(3, 663)_ = 36.59, *p* < 0.001, *η*^*2*^_*p*_ = 0.142) confirmed all the above-mentioned post-hoc comparisons in both groups and also that YA performed better than OA in all filtering conditions (all *p* < 0.001) except for hybrid stimuli, for which the comparison was not significant. Furthermore, the interaction between Group and Orientation (*F*_(1, 221)_ = 36.54, *p* < 0.001, *η*^*2*^_*p*_ = 0.142) confirmed a better performance for Upright than for Upside-down orientation in both groups (*p* < 0.001). Finally, it also revealed that the performance of YA was better than OA for both Upright and Upside-down orientations (*p* < 0.001).

The significant interaction between Emotion and Orientation (*F*_(1, 221)_ = 58.57, *p* < 0.001, *η*^*2*^_*p*_ = 0.21) confirmed a better performance for the Upright compared to the Upside-down condition with both Angry and Happy emotions, together with a better performance for Happy than for Angry faces presented both Upright and Upside-down (all *p* < 0.001). Emotion x Filtering interaction (*F*_(3, 663)_ = 60.43, *p* < 0.001, *η*^*2*^_*p*_ = 0.215), confirming higher Pr for Happy than for Angry faces in all filtering conditions, also showed that only for the Happy expression, the performance was significantly different between each filtering condition (Happy: Unfiltered > HSF > LSF > Hybrid), but no difference emerged between Unfiltered and HSF for Angry expression (Angry: Unfiltered = HSF > LSF > Hybrid). Also, the Filtering x Orientation interaction was significant (*F*_(3, 663)_ = 80.21, *p* < 0.001, *η*^*2*^_*p*_ = 0.27), confirming a better performance for Upright than for Upside-down orientation in all filtering conditions (all *p* < 0.001), as well as a lower performance within each orientation for Hybrid than LSF, LSF compared to HSF, and for HSF than for Unfiltered stimuli (Unfiltered > HSF > LSF > Hybrid; *p* < 0.001), except for the non-significant comparison between Unfiltered and HSF in the Upright orientation.

The three-way interaction among Group, Emotion, and Orientation (*F*_(1, 221)_ = 13.03, *p* < 0.001, *η*^*2*^_*p*_ = 0.056) showed that the performance of the YA group was better than that of the OA group in all conditions and that in both groups the performance was better for Upright compared to Upside-down orientation, except for YA in Happy condition which failed to reach significance (Upright vs Upside-down: *p* = 0.058). The significant interaction among Group, Emotion, and Filtering (*F*_(3, 663)_ = 3.10, *p* = 0.026, *η*^*2*^_*p*_ = 0.014) showed a better performance of YA compared to OA in all conditions (*p* < 0.001), except for Hybrid Angry and Hybrid Happy faces, in which comparisons were not significant. Furthermore, for both YA and OA groups and Angry and Happy stimuli, the performance was not different between Unfiltered and HSF stimuli. Still, it was higher in these conditions compared to the LSF and higher for the LSF compared to the Hybrid condition (i.e., Unfiltered = HSF > LSF > Hybrid), with this latter comparison being not significant only for YA with Happy faces. The interaction among Group, Orientation, and Filtering was significant (*F*_(3, 663)_ = 13.49, *p* < 0.001, *η*^*2*^_*p*_ = 0.058). Post-hoc comparisons confirmed a better performance of the YA than the OA group in both orientations and for all Filtering conditions, except for Hybrid stimuli which did not reach statistical significance. Emotion, Orientation, and Filtering significantly interacted (*F*_(3, 663)_ = 32.84, *p* < 0.001, *η*^*2*^_*p*_ = 0.13), confirming Unfiltered > HSF > LSF > Hybrid for both Angry and Happy faces presented in Upright orientation, and only for Angry faces presented in Upside-down orientation. For Happy stimuli in Upside-down orientation, there was no difference between Unfiltered and HSF filtering (Unfiltered = HSF > LSF > Hybrid).

**Fig. 3:**
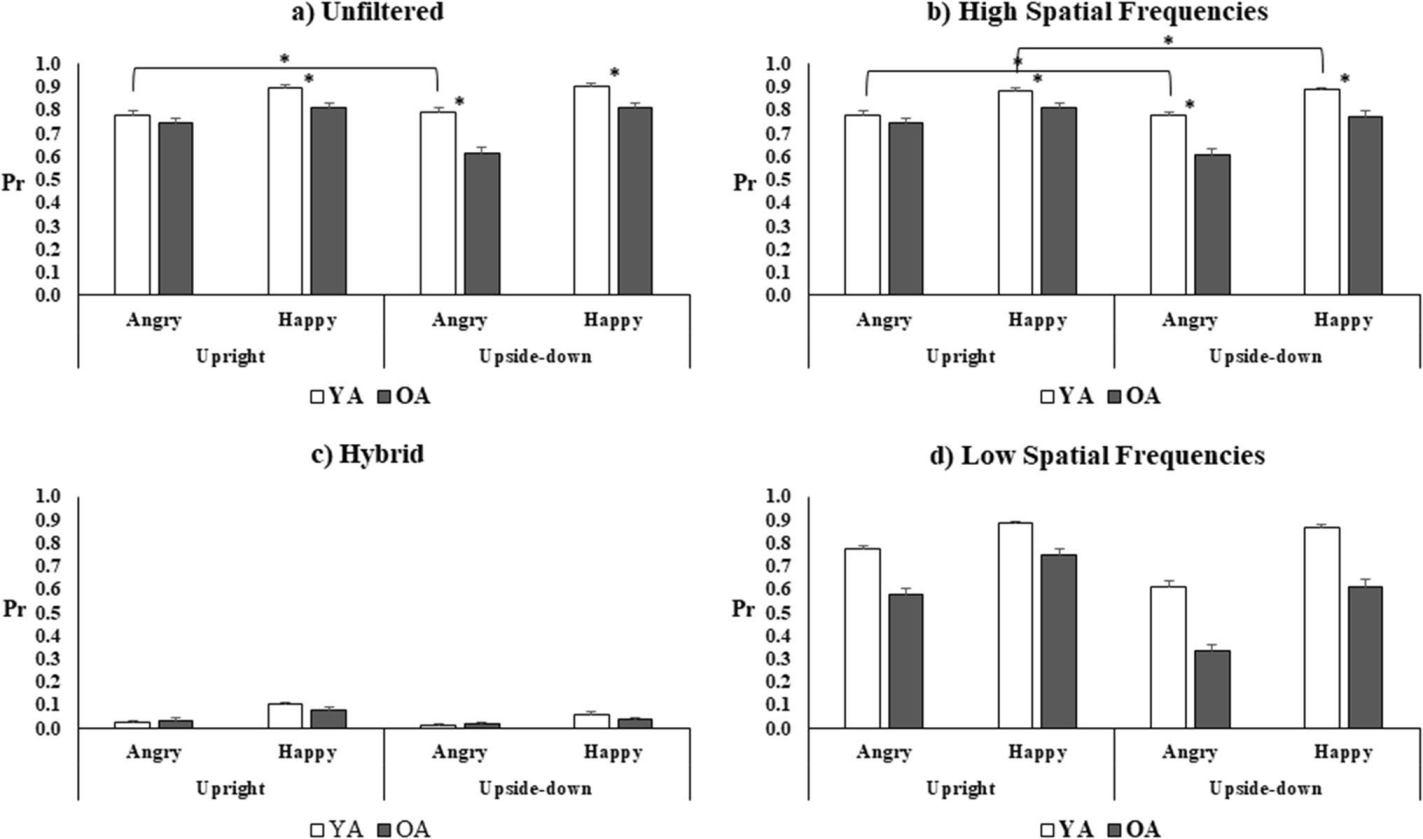
Results of the four ANOVAs carried out on Pr scores, showing the interactions among Group (Younger Adults: YA, Older Adults: OA), Emotion (Angry, Happy), and Orientation (Upright, Upside-down) for each filtering condition: **a** Unfiltered, **b** filtered at High Spatial Frequencies, **c** hybrid, **d** filtered at Low Spatial Frequencies. Panels **a** and **b** represent the significant interaction, whereas panels **c**, **d** show no significant interactions. Bars represent standard errors, and asterisks show significant post-hoc comparisons (to note that, even if not showing by asterisks, all comparisons between Happy and Angry are significant for both Unfiltered and HSF conditions)

Finally, the interaction among all four factors was significant (*F*_(3, 663)_ = 12.97, *p* < 0.001, *η*^2^_*p*_ = 0.055). To better understand this four-way interaction, four different ANOVAs were carried out, one for each level of Filtering, using Group as the between-subject factor and Emotion and Orientation as within-subject factors. The interaction between Group, Emotion, and Orientation was significant for Unfiltered (*F*_(1, 221)_ = 37.41, *p* < 0.001, *η*^2^_*p*_ = 0.14; Fig. 3a) and for HSF stimuli (*F*_(1, 221)_ = 22.09, *p* < 0.001, *η*^2^_*p*_ = 0.09; Fig. 3b), but it was not significant for LSF stimuli (see Fig. 2c) and for Hybrid stimuli (Fig. 3d).

In both Unfiltered and HSF ANOVAs, post-hoc comparisons confirmed a better performance of the YA than the OA group for Happy faces presented both Upright and Upside-down and Angry faces Upside-Down. Moreover, in both ANOVAs, in all conditions and groups, the performance was better for Happy than Angry faces. Post-hoc tests in the Unfiltered ANOVA (Fig. 3a) showed that in the OA group, the performance was significantly better when Angry faces were presented Upright instead of Upside-down (*p* < 0.001). In the HSF ANOVA (Fig. 3b), post-hoc tests revealed that for the OA group, the performance was better for Upright compared to Upside-down orientation for both Angry (*p* < 0.001) and Happy faces (*p* = 0.001).

## Discussion

The present study investigated the relationship between implicit vs. explicit emotion processing and ageing (Schiller et al. 2024). We exploited the spatial filtering technique to indirectly investigate the functioning of the subcortical route in subliminal emotion processing through a go/no-go emotional task. Starting from previous evidence (Petro et al. 2021), we expected that ageing would impair the subcortical route and it would enhance the functioning of the cortical route, leading to (i) a worse performance in detecting emotional faces in older compared to younger participants, when stimuli were presented at LSF and hybrid (with the emotional content presented in the LSF and hidden by a neutral expression superimposed at HSF). In contrast, we expected (ii) no difference between the two age groups for unfiltered stimuli and stimuli filtered at HSF because this processing would activate the cortical route, which is supposed to be strongly activated in ageing (Petro et al. 2021). Furthermore, we also expected that (iii) all stimuli presented rotated 180° about the vertical axis (i.e., upside-down) would lead to worse performance in general, with either a possible specific effect in ageing, due to the fact that face-inversion is based on a global analysis, which in turn should be based on the subcortical route; or a similar performance in the two age groups starting from other evidence suggesting that global analysis is intact in the elderly (Boutet and Faubert 2006). Finally, (iv) starting from the positivity bias described in ageing (Barber et al. 2022; Carstensen et al. 2006; Charles 2010; Mather 2016; Mather and Knight 2005; Reed et al. 2014), we expected a lower performance of older adults to be evident only for the negative emotion (i.e., anger), mainly when processed through the subcortical route (LSF and hybrids), but either no age difference or better performance in older adults compared to younger participants was hypothesised for the positive emotion (i.e., happiness).

Results showed an overall better performance for younger compared to older participants, which would be due to a general perceptual and cognitive decline in ageing (Fisk and Warr 1996; Meinhardt-Injac et al. 2014), as revealed by the analyses carried out on Pr, which is a synthetic and informative value in such a paradigm, being calculated as the difference between hits and false alarms (Snodgrass and Corwin 1988). In this frame, it is useful to underline that only hits and false alarms were considered in the present study without considering response times, specifically due to the well-known slowdown in ageing (Ruffman et al. 2008). In fact, we did not required participants to respond as soon as possible (so that response times were not considered as dependent variable). Moreover, the go/no-go paradigm was chosen to favour the participation of older people, hypothesizing that only one response (and one response key) would be preferred to more complex response options, especially for people who are not confident with electronical devices. Nevertheless, a group difference in Pr scores emerged for unfiltered, LSF, and HSF stimuli, as revealed by the interaction between group and filtering, with only hybrid stimuli not showing a worse performance in older adults. This absence of age difference for hybrid faces seems to be due to a low performance by younger participants rather than to a high performance by older participants, and this can be attributable to the peculiar composition of hybrid stimuli: as specified above, in fact, in hybrid faces, the emotional content presented at LSF is “hidden” by the neutral content presented at HSF. Since the specific task used here is an “explicit” categorisation task (emotional vs neutral), we can speculate that the conscious output of the cortical route (neutral HSF) was predominant in driving the explicit response required, overriding the activity of the subcortical route (emotional LSF). In this context, the present results align with the pioneering evidence described by Laeng and colleagues with hybrid faces (Laeng et al. 2010), showing that participants labelled all hybrid emotional stimuli as neutral when an explicit categorisation was required. Coming back to our experimental hypotheses, we found support (i) for the absence of an age difference for hybrid stimuli, but we found support neither for the expected absence of age difference for LSF stimuli, (ii) nor for the expected similar performance across different age groups for stimuli presented unfiltered and filtered at HSF. This pattern of results is not in line with the expected weaker activity in ageing, specifically for the subcortical route. Still, it seems to support a general worsening in emotion detection in ageing. Furthermore, interactions confirmed that the absence of age differences emerged for hybrid stimuli presented both with a happy and an angry expression (group × emotion × filtering) and for hybrid stimuli presented both upright and upside-down (group × orientation × filtering), confirming an overall inability to explicitly categorise the emotional content hidden in hybrid faces in a go/no-go task.

Concerning the third hypothesis, results confirmed the expected face-inversion effect (Prete et al. 2015c, d), but also, in this case, no age difference emerged, with worse performance for stimuli presented upside-down than upright in both younger and older participants, for both happy and angry faces, and in all filtering conditions. This result, confirming that faces are processed holistically, does not support a specific impairment for upside-down faces in ageing, confirming the evidence according to which global analysis of faces remains the same throughout the lifespan (Boutet and Faubert 2006).

Lastly, a positivity bias specific to older participants was hypothesised (Barber et al. 2022; Mather 2016): the present results confirmed a better performance for happy compared to angry faces, but (iv) no ageing effect was found since both groups showed a better performance with the positive than the negative emotion. This finding can be ascribed to a general facilitation often described for happiness compared to the other basic emotions (Leppänen and Hietanen 2004). A peculiar interaction between emotion and filtering revealed that, while for happy faces the performance was statistically different among each level of filtering (e.g., unfiltered > HSF > LSF > hybrid), for angry faces there was no difference between LSF and hybrid stimuli. This pattern is in line with the idea that happiness is a “distal” emotion based on LSF processing, whereas anger would be a “proximal” emotion based on the HSF (Smith and Schyns 2009). Thus, only for happiness the performance was better for LSF alone than for the “mixed” (emotional and neutral) hybrid condition.

Finally, the interaction among all the factors clarifies and substantially confirms all the interactions discussed above. Four analyses were carried out to shed more light on these results, splitting data according to the specific filtering condition. Results confirmed no significant interaction among group, emotion, and orientation for hybrid stimuli and—surprisingly—for stimuli filtered at LSF. This evidence confirms that the present results do not support the hypothesised lower activity of the subcortical route in ageing because no age difference emerged in both the conditions in which LSF conveyed an emotional content (LSF and hybrid). The fact that such an age difference, with younger participants outperforming older participants, is present in both unfiltered and HSF conditions, suggests a stronger functioning of the cortical route in younger compared to older participants. Only in the unfiltered condition, the performance of the older adults did not differ for happy faces presented upright vs. upside-down, possibly showing a resistance to the face-inversion effect only for happiness, which remains the emotion leading to better performance. However, the fact that angry faces presented upright in both unfiltered and HSF conditions did not reveal age differences seems to confirm that when a “proximal” emotion is shown (HSF; (Smith and Schyns 2009) and faces are presented in canonical orientation (upright), older participants show the same performance as younger ones, partially confirming the hypothesis of a preserved ability to process emotions by means of the cortical route.

The study contains some critical aspects that should be fully considered in future works. Primarily, the evidence collected here is an indirect measure of the functioning of the dual route model, indeed neuroscientific data must be collected to prove the activity of the cortical vs subcortical route at different ages. Furthermore, this is the first-time hybrid faces were administered to participants older than 65. Therefore, all data collected in previous research involving young participants are considered reference data here, but no direct evidence exists about the cerebral processing of hybrid faces in ageing. This is also a strength of the work, but subliminal emotion processing of these stimuli in older adults, as shown with younger observers (Laeng et al. 2010; Prete et al. 2015a), should be proven. However, the fact that the hybrid condition is the only condition in which no difference emerges between the two age groups indirectly shows that these stimuli are processed similarly in younger and older adults. Another point to be stressed is the online administration of the paradigm. When images are filtered at different spatial frequencies, the exact distance the observer is positioned with respect to the stimulus is crucial for the exact range of SF viewed. Regarding this point, in the instructions given before the task, we specifically stressed the importance of maintaining the required distance from the screen, but it must be acknowledged that different the devices used prevent us to maintain a control over the specific features of the computer used (e.g., screen size, brightness and so on). Moreover, we recruited a large sample of participants to compensate for possible issues in this regard. Finally, it must be highlighted that only one positive and one negative emotion were used in the present study, so the results are not generalisable to all emotions. We selected happiness and anger as they are the two facial expressions that received the most extreme friendliness judgments in the original study by Laeng and colleagues (Laeng et al. 2010), but further evidence with different emotions should be described to reach a general conclusion in this domain.

To conclude, the performance in a go/no-go emotional task was lower for older compared to younger participants, but at a deeper analysis, this difference was specific only for some comparisons. We found that independently of low or high spatial filtering, younger adults outperformed older adults so that no specific conclusion about the outcome of the cortical vs. subcortical route emerged clearly. However, based on previous evidence, we speculate that emotional expressions contained in hybrid faces are processed implicitly (Laeng et al. 2010, 2013; Prete et al. 2015a, 2014, 2018, Prete et al. 2015c), so in the go/no-go task used here a floor effect emerged in both age groups when positive and negative hybrid stimuli had to be explicitly recognised as emotional vs. neutral. The results of the present study also confirmed that faces are processed holistically, showing that the expected face-inversion effect persists at any age (Boutet and Faubert 2006) and that happiness is the most straightforward emotion to be recognised (Leppänen and Hietanen 2004). No positivity bias specific to ageing emerged from the present study (Reed and Carstensen 2012; Zebrowitz et al. 2015). Still, data confirmed a better performance in processing happiness compared to anger in both younger and older participants. We suggest that no specific effects of ageing on the activity of the dual route implicated in emotion processing are evident (Johnson 2005; LeDoux 1996), but a general decrement in emotion detection emerged through the go/no-go task, confirming a general perceptual and/or cognitive worsening in the elderly, without compromising global analysis or a specific emotion. Although further studies, mainly involved neuroscientific methodologies, are needed to directly show the activity of the two routes in ageing, the present results are the first evidence of a normal and expected worsening of perceptual processing of emotions with ageing, without a specific impairment in the functioning of both the cortical-conscious and the subcortical-unconscious route.
